# Structural mechanism underlying primary and secondary coupling between GPCRs and the Gi/o family

**DOI:** 10.1038/s41467-020-16975-2

**Published:** 2020-06-22

**Authors:** Hee Ryung Kim, Jun Xu, Shoji Maeda, Nguyen Minh Duc, Donghoon Ahn, Yang Du, Ka Young Chung

**Affiliations:** 1 School of Pharmacy, Sungkyunkwan University, 2066 Seobu-ro, Jangan-gu Suwon, 16419 Republic of Korea; 2 Beijing Advanced Innovation Center for Structural Biology, School of Medicine, Tsinghua University, Beijing, 100084 China; 3 Department of Molecular and Cellular Physiology, School of Medicine, Stanford University, 279 Campus Drive, Stanford, CA 94305 USA; 4 Division of Precision Medicine, Research Institute, National Cancer Center, 323 Ilsan-ro, Ilsandong-gu, Goyang 10408 Republic of Korea; 5 School of Life and Health Sciences, Kobilka Institute of Innovative Drug Discovery, Chinese University of Hong Kong, 2001 Longxiang Ave, Shenzhen, Guangdong 518172 China

**Keywords:** Structural biology, Cell signalling, Molecular biology

## Abstract

Heterotrimeric G proteins are categorized into four main families based on their function and sequence, Gs, Gi/o, Gq/11, and G12/13. One receptor can couple to more than one G protein subtype, and the coupling efficiency varies depending on the GPCR-G protein pair. However, the precise mechanism underlying different coupling efficiencies is unknown. Here, we study the structural mechanism underlying primary and secondary Gi/o coupling, using the muscarinic acetylcholine receptor type 2 (M2R) as the primary Gi/o-coupling receptor and the β_2_-adrenergic receptor (β_2_AR, which primarily couples to Gs) as the secondary Gi/o-coupling receptor. Hydrogen/deuterium exchange mass spectrometry and mutagenesis studies reveal that the engagement of the distal C-terminus of Gαi/o with the receptor differentiates primary and secondary Gi/o couplings. This study suggests that the conserved hydrophobic residue within the intracellular loop 2 of the receptor (residue 34.51) is not critical for primary Gi/o-coupling; however, it might be important for secondary Gi/o-coupling.

## Abstract (web summary)

G protein-coupled receptors (GPCRs) can couple to more than one G protein subtype, and the coupling efficiency varies depending on the GPCR-G protein pair. Here authors use hydrogen/deuterium exchange mass spectrometry and mutagenesis to study the structural mechanism underlying primary and secondary Gi/o coupling.

## Introduction

G protein-coupled receptors (GPCRs) are the largest receptor superfamily that perceive extracellular signals, including light, smell, taste, hormones, and neurotransmitters^1^. Due to their critical functions in physiology and pathology, GPCRs are good therapeutic targets, and one third of approved medicines acts on GPCRs. Thus, it is important to understand the precise signaling mechanism of GPCRs for our fundamental knowledge on the cellular signaling and for the development of better GPCR-targeting therapeutics.

GPCRs propagate extracellular signals into cells by coupling with the heterotrimeric guanine-nucleotide-binding regulatory proteins (G proteins)^2^. The heterotrimeric G proteins are formed by α, β, and γ subunits; in the basal state, Gα is occupied by guanosine diphosphate (GDP) and interacts with Gβγ subunits. An agonist-activated receptor interacts with a G protein, which triggers the exchange of GDP for guanosine triphosphate (GTP), followed by dissociation of the Gα subunit from the receptor and Gβγ subunits^2,3^. The GTP-bound Gα subunit or Gβγ subunits interact with and regulate downstream effector proteins. Based on their downstream function and sequence, the Gα subunits are grouped into four families, Gαs (Gαs and Gα_olf_), Gαi/o (Gαi1, Gαi2, Gαi3, Gαo, Gαt1, Gαt2, Gαt3, and Gαz), Gαq/11 (Gαq, Gα11, Gα14, and Gα16), and Gα12/13 (Gα12 and Gα13)^1^.

Approximately 800 GPCRs have been identified in humans. Previous studies have reported that many GPCRs exhibit promiscuous GPCR-G protein coupling; i.e., a single receptor can interact with more than one G protein subtype^4–6^. One receptor often couples to more than one Gα isoform within the same family due to high sequence similarity^5^. For example, the serotonin 1 A receptor interacts with three Gi/o family proteins, Gi2, Gi3, and Go^5^. Moreover, several receptors can couple to different G protein families; the α_2_- and β_2_-adrenergic receptors (α_2_AR and β_2_AR) interact with Gs and Gi/o^7,8^, the PAR1 receptor with Gi/o and G12/13^9^, and the melanin-concentrating hormone receptor 1 with Gi/o and Gq/11^10^.

The interaction efficiency and/or binding kinetics of one receptor to different G proteins often differ. The most prominent coupling, which shows the highest coupling efficiency with fast kinetics, is referred to as ‘primary coupling’. The minor coupling, which shows lower coupling efficiency and/or slower kinetics, is referred to as ‘secondary coupling’^11,12^. The known primary and secondary GPCR-G protein pairs have been summarized in the IUPHAR/BPS Guide to Pharmacology^11^ and GPCRdb (gpcrdb.org) (Supplementary Fig. 1a).

Several biochemical/biophysical studies have revealed the conformational dynamics and high-resolution structures of G proteins in various states^1,3^. These structures of GPCR-G protein complexes reveal the interactions between GPCRs and nucleotide-free G proteins^13–15^. The X-ray crystal structure of the β_2_AR-Gs complex^15^ and the cryo-electron microscopy (cryo-EM) structure of the muscarinic acetylcholine receptor type 2-GoA (M2R-GoA) complex^16^ are shown in Supplementary Fig. 2. The major binding interface between a receptor and a G protein for both structures is between the C-terminal part of α5 of the Gα subunit and the cytosolic core of the receptor formed by transmembrane domains (Supplementary Fig. 2). The distal C-terminus of Gα (the so-called ‘wavy hook’) (Supplementary Fig. 2c, green dotted line)^17,18^ forms an additional α-helical-turn as an extension of α5 when it interacts with a receptor (Supplementary Fig. 2c). Another interface is the intracellular loop 2 (ICL2) of the receptor interacting with the hydrophobic pocket of Gα formed by hydrophobic residues at the αN/β1 hinge, β2/β3 loop, and α5 (Supplementary Fig. 2a, blue dotted box). This interaction appears to be weaker for M2R-GoA structure compared with β_2_AR-Gs, which is discussed below.

Recently, using a combination of pulsed hydrogen/deuterium exchange mass spectrometry (HDX-MS), pulsed hydroxyl radical footprinting mass spectrometry (HRF-MS), and mutational studies, we proposed a model that delineates the sequential events during β_2_AR-Gs coupling, the primary GPCR-Gs pair^19^. In brief, the C-terminal region of Gαs initially associates with the β_2_AR followed by interaction of ICL2 with the hydrophobic pocket within Gαs, which is the key step for GDP release (see below). Stable helix formation of the Gαs wavy hook occurs slowly after GDP release.

Although there has been a great progress in understanding the structural mechanism of GPCR-G protein coupling as described above, the structural mechanism of different coupling efficiencies observed from the primary and the secondary coupling has not been clearly elucidated. In the current study, we took advantage of HDX-MS and investigate the structural mechanism that differentiates primary and secondary GPCR-Gi/o coupling using the M2R and the β_2_AR as model GPCRs, and Gi3 and GoA as model Gi/o proteins. The data suggest that the C-terminus of Gαi/o differentiates primary and secondary Gi/o-coupling and that residue 34.51 of the receptor is not important for the primary Gi/o coupling.

## Results

### M2R- and β_2_AR-induced GDP release from Gi/o proteins

The IUPHAR/BPS Guide to Pharmacology^11^ specifies that the M2R transduces signals primarily through Gi/o family and secondarily through Gs and Gq/11 families, and the β_2_AR transduces signals primarily through Gs family and secondarily through Gi/o family (Supplementary Fig. 1b). Especially, the coupling efficiencies of the β_2_AR with Gs or Gi/o families have been studied extensively both in vitro and in vivo^20–22^, and the coupling efficiencies of the M2R with Gs, Gi/o, and Gq/11 families have been studied in vivo^23,24^.

**Fig. 1:**
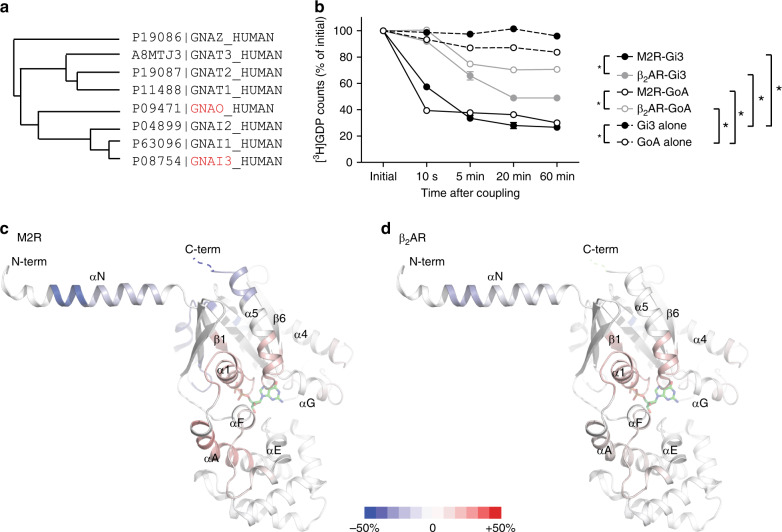
GDP release and HDX-MS analysis of different GPCR-G protein pairs. **a** Phylogenic tree of Gαi/o families. The phylogenic tree is generated based on the protein sequences provided by UNIPROT database (uniprot.org). The uniprot ID is indicated in front of the protein names. **b** GDP release profiles of GoA and Gi3, induced by the M2R or the β_2_AR. * Indicates statistical differences between groups analyzed by one-way ANOVA test (*p* < 0.05). Error bars represent mean ± S.E.M. of three independent experiments. Source data are provided as a Source data file. **c**, **d** HDX level changes of GαoA after 3 h of incubation with the M2R (**c**) or the β_2_AR (**d**). The changes in HDX are color-coded on the X-ray crystal structure of Gαi1 (PDB 1GP2). The deuterium uptake plots are provided in Supplementary Fig. 4a.

To confirm the different coupling efficiencies of the two different receptors (i.e., primary vs. secondary) for Gi/o proteins, we analyzed receptor-mediated GDP release using purified proteins in vitro. Herein, we used Gi3 and GoA as model Gi/o proteins (Fig. 1a) as the β_2_AR-Gi3 interaction has been well-characterized in a previous study^21^, and the high-resolution structure of M2R-GoA has been resolved by cryo-EM^16^. To achieve optimal β_2_AR-Gi/o coupling, we used micelles composed of negatively charged lipids (DDM with POPE at a ratio of 5:1) as the previous study had shown that the charge state of the lipid surrounding the β_2_AR affects the efficiency of Gi/o coupling^21^. As expected, the M2R coupled more efficiently to both Gi3 and GoA than the β_2_AR, as demonstrated by faster GDP release, with higher efficacy (Fig. 1b) confirming that M2R is primary Gi/o-coupled receptor and β_2_AR as secondary Gi/o-coupled receptor. Notably, our previous study showed that the β_2_AR induced almost complete GDP release from Gs within 10 s^19^, confirming that the β_2_AR primarily couples to Gs.

### M2R- or β_2_AR-induced HDX profile changes in GαoA

Previously, we analyzed the conformational differences between GDP-bound Gαs and β_2_AR-bound nucleotide-free Gαs using HDX-MS^25^; HDX levels near the nucleotide-binding pocket were higher in β_2_AR-bound nucleotide-free Gαs than in GDP-bound Gαs, which reflects GDP release and increased structural dynamics in this region (Supplementary Fig. 3). The C-terminal part of α5 of Gαs displayed a lower HDX level in β_2_AR-bound nucleotide-free Gαs than in the GDP-bound Gαs (Supplementary Fig. 3) reflecting a stable helix formation and insertion into the receptor cytosolic core (described in Supplementary Fig. 2c).

To understand the structural mechanism of different GDP release efficiencies between M2R-Gi/o and β_2_AR-Gi/o pairs, here we analyzed HDX levels of GαoA in GDP-bound GoA heterotrimer alone and in GoA heterotrimer after M2R or β_2_AR co-incubation (Fig. 1c, d and Supplementary Fig. 4a). The co-incubation of GoA with the M2R or the β_2_AR did not affect HDX profiles of Gβγ subunits (Supplementary data) implying that Gβγ subunits do not undergo significant conformational changes upon complex formation with the M2R or the β_2_AR. When GoA was incubated with its primary coupling receptor M2R, we detected decreased HDX at the C-terminal part of α5 (Fig. 1c and Supplementary Fig. 4a, peptides 341‒348 and 349‒353), which suggests decreased dynamics and/or exclusion from the buffer. We detected increased HDX near the nucleotide binding pocket (Fig. 1c and Supplementary Fig. 4a, peptides 37‒52, 268‒275, and 324‒333), which suggests GDP release and/or increased dynamics. We also detected increased HDX between Ras-like and α-helical domains (Fig. 1c and Supplementary Fig. 4a, peptides 53‒61, 276‒285, and 289‒298) and a few peptides from the α-helical domain (AHD) (Fig. 1c and Supplementary Fig. 4b, peptides 65‒77 and 161‒173), which could be attributed to domain opening and dynamic movement of AHD^26^. These results are consistent with those of the previous HDX-MS study that analyzed β_2_AR-Gs complex (Supplementary Fig. 3a)^25^. We detected decreased HDX at αN (Fig. 1c and Supplementary Fig. 4a, peptides 14‒18 and 19‒29), which was not detected in β_2_AR-Gs complex due to lack of identified peptides in this region; the decreased HDX at αN may reflect the interaction of this region with the M2R (Supplementary Fig. 2b) and/or the blockage of this region from the buffer by micelles surrounding the receptor.

When GoA was incubated with the β_2_AR, a secondary coupling receptor, the HDX profile changes were similar in most regions except the C-terminal part of α5 (Fig. 1c, d). For instance, we detected increased HDX near the nucleotide-binding pocket and AHD, and decreased HDX at αN, although to a lesser extent than the M2R-GoA pair (Fig. 1d and Supplementary Fig. 4a). However, surprisingly, the C-terminal part of GαoA did not show HDX profile changes (Fig. 1d and Supplementary Fig. 4a, peptides 341‒342 and 349‒353), which implies that the C-terminal part of GαoA may not form the stable helix upon β_2_AR-GoA coupling and/or may not be deeply inserted into the receptor core.

### Time-resolved HDX during M2R- and β_2_AR-Gi/o coupling

The HDX profile changes shown in Fig. 1 are comparisons between before and after 3 h of co-incubation of GoA with the receptors. Three hours of co-incubation of the receptor and GoA is sufficient for completion of GDP release and formation of the final receptor-GoA complex^16,21^. Therefore, the data in Fig. 1 do not reflect transient conformational changes during coupling.

Recently, we performed HDX-MS in a pulsed manner to gain insight into time-resolved conformational changes during primary GPCR-Gs coupling using β_2_AR-Gs pair as model system^19^. In this previous study, the β_2_AR and Gs were sampled before co-incubation, and after 10 s, 5 min, 20 min, 60 min, 90 min, 110 min, 150 min, and 180 min of co-incubation. The sampled proteins were then pulsed with deuterated buffer for 10 or 100 s^19^. This approach provides the HDX profiles of the indicated time points, which reflect the conformational states of the β_2_AR and Gs at the sampled time points (Supplementary Fig. 5a). Pulsed HDX-MS analysis of β_2_AR-Gs revealed HDX profile change at ICL2 of the β_2_AR within 10 s of co-incubation (Supplementary Fig. 5a, peptides 133‒144) whereas the HDX profile change continued until 110 min at the N-terminal part of ICL3 (Supplementary Fig. 5a, peptide 223–240), which suggests that ICL2 undergoes conformational changes faster than ICL3 upon coupling to Gs. Analysis of Gαs detected rapid HDX profile changes at the nucleotide-binding pocket (Supplementary Fig. 5a, peptides 49‒59 and 367‒371) and slow and prolonged HDX profile changes at the C-terminus of α5 (Supplementary Fig. 5a, peptide 382‒390)^19^. We confirmed that GDP release occurs within 10 s of co-incubation with the β_2_AR indicating that the rapid HDX profile changes at the nucleotide-binding pocket was mainly due to GDP release^19^. Interestingly, the prolonged HDX profile changes at the C-terminus of α5 suggested that this region undergoes prolonged conformational changes even after GDP release^19^.

**Fig. 2:**
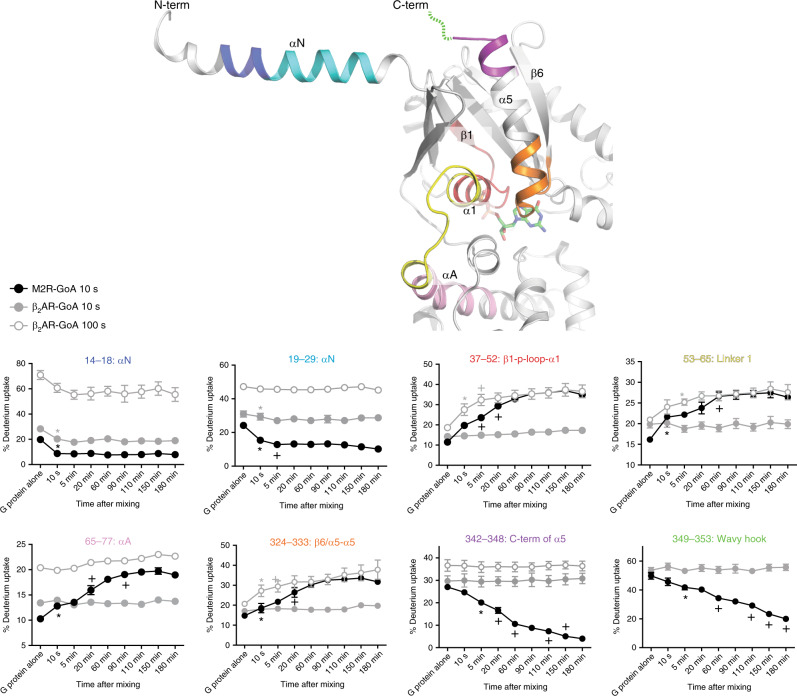
Time-resolved HDX profiles of GαoA during M2R-GoA and β_2_AR-GoA coupling. The selected analyzed peptides are color-coded on the X-ray crystal structure of Gαi1 (PDB 1GP2) and on the titles of deuterium uptake graphs. Statistically significant changes during coupling were analyzed by repeated-measures ANOVA (rANOVA). To compare two different time points, a two-tailed paired Student’s t-test was performed, and *p* < 0.05 was considered as statistically significant. * Indicates the first time point that showed a statistical difference compared with before co-incubation. + Indicates the first time point that showed a statistical difference from previously marked (either * or +) time point. Error bars represent mean ± S.E.M of three independent experiments. Please note that the data is plotted using a non-linear/non-logarithmic scale.

In the current study, we adopted the same strategy to investigate whether the C-terminal part of Gαi/o does ever form a stable helix and/or is inserted into the receptor cytosolic core, during the time-course of β_2_AR-Gi/o coupling (Fig. 2 and Supplementary Fig. 5b). We used 10 s deuterium pulse for M2R-Gi/o coupling and 10 and 100 s deuterium pulses for β_2_AR-Gi/o coupling because 10 s deuterium pulse was not sufficient to observe HDX differences between Gi/o alone and Gi/o co-incubated with the β_2_AR, for a few peptides (for example, peptides 37‒52, 53‒61, 268‒275, and 324‒333 in Supplementary Fig. 4a). Again, we did not detect any HDX profile changes of Gβγ subunits during the co-incubation time course (Supplementary data).

When GoA or Gi3 was co-incubated with the M2R, the HDX profile change at αN was initiated within 10 s and completed within 10 s to 5 min (peptides 14‒18 and 19‒29 for GαoA in Fig. 2 and peptides 6‒18 and 19‒33 for Gαi3 in Supplementary Fig. 5b). The HDX profile changes of αN showed similar results when Gi3 or GoA was incubated with the β_2_AR (Fig. 2 and Supplementary Fig. 5b). However, the previous study with the β_2_AR and Gs reported that HDX profile at αN of Gαs increased within 10 s of incubation with the β_2_AR (Supplementary Fig. 5a) suggesting that the conformational changes at αN may differ between GPCR-Gs and GPCR-Gi/o coupling.

The HDX profile change near the nucleotide-binding pocket was initiated within 10 s and completed within 5‒20 min in M2R-Gi3/GoA pairs (peptides 37‒52 and 324‒333 for GαoA in Fig. 2 and peptides 37‒52 and 324‒334 for Gαi3 in Supplementary Fig. 5b). Interestingly, GDP release was completed within 10 s to 5 min for M2R-Gi/o coupling (Fig. 1b), which was faster than the changes in HDX-MS profile near the nucleotide-binding pocket. This may reflect further conformational changes after GDP release; the initial HDX profile changes within 10 s to 5 min were due to GDP release, while the later HDX profile changes observed at 20 min were due to additional conformational changes after GDP release. However, we cannot exclude the possibility that these discrepancies between GDP release and HDX-MS profile change kinetics could also be due to different experimental conditions between the two assay systems such as protein concentration (see the Methods for details).

When Gi3 or GoA was incubated with the β_2_AR, the HDX profile changes near the nucleotide-binding pocket were initiated within 10 s and completed within 10 s to 5 min, which was faster than M2R-Gi3/GoA pairs (Fig. 2 and Supplementary Fig. 5b). The GDP release was initiated within 5 min and completed within 20 min of co-incubation of the β_2_AR and Gi3/GoA (Fig. 1b), and the discrepancies between GDP release and HDX-MS profile change kinetics could be due to different experimental conditions between the two assay systems; the coupling may be slower in the GDP release assay probably due to lower protein concentrations. Overall, it is tempting to propose that the nucleotide-binding pocket undergoes further conformational changes after GDP release in M2R-Gi3/GoA coupling whereas the conformational changes near the nucleotide-binding pocket in β_2_AR-Gi3/GoA pairs are smaller and quicker probably due to the lack of further conformational changes after GDP release.

The HDX profile of the C-terminal part of GαoA continued to decrease until 150‒180 min during M2R-GoA coupling (Fig. 2, peptides 342‒348 and 349‒353), which is similar to what we observed with β_2_AR-Gs coupling^19^ (Supplementary Fig. 5a, peptides 382‒390). On the other hands, during M2R-Gi3 coupling, the HDX profile change at the C-terminal part of Gαi3 was initiated within 10 s and continued to decrease until 20 min (Supplementary Fig. 5b, peptides 342‒348 and 349‒353). The molecular mechanism underlying the different HDX profile change kinetics at the C-terminal part of GαoA and Gαi3 needs further investigation. Interestingly, when incubated with the β_2_AR, the C-terminal part of GαoA or Gαi3 never showed decreased HDX at any time point during coupling (Fig. 2 and Supplementary Fig. 5b).

In summary, the M2R-induced HDX profile changes in GαoA or Gαi3 were similar to what we observed for the β_2_AR-induced HDX profile changes in Gαs except at the αN. However, β_2_AR-Gi/o complex showed no HDX-MS profile change at the C-terminal part of Gαi/o, which implied that this region of Gαi/o does not form stable helices during β_2_AR-Gi/o coupling and/or may not be deeply inserted into the cytoplasmic core of the receptor.

### The role of the wavy hook for Gi/o coupling

As the C-terminal part of Gαi/o displayed the most differences in HDX-MS profiles between M2R-Gi/o and β_2_AR-Gi/o coupling, we hypothesized that the C-terminal part of Gαi/o differentiates between primary and secondary GPCR-Gi/o coupling.

**Fig. 3:**
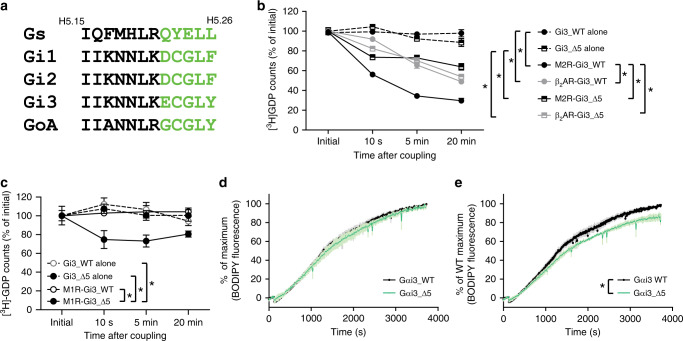
The role of Gαi3 wavy hook in primary and secondary Gi coupling. **a** Sequence alignment of the C-terminal part of Gαs and Gαi/o proteins. **b** GDP release profiles of Gi3_WT and Gi3_Δ5 induced by the M2R or the β_2_AR. **c** GDP release profiles of Gi3_WT and Gi3_Δ5 induced by the M1R. For (**b**) and (**c**), * indicates statistical differences between groups analyzed by one-way ANOVA test (*p* < 0.05). Error bars represent mean ± S.E.M of at least three independent experiments (*n* = 3–6). **d** BODIPY fluorescence presented as percent (%) of maximum of each sample. **e** BODIPY fluorescence represented as percent (%) of WT Gαi3 maximum. Error bars (gray for WT Gαi3 and light green for Gαi3_Δ5) represent mean ± S.E.M of three independent experiments. * Indicates statistical differences between groups analyzed by a two-tailed Student’s *t* test (*p* < 0.05). Source data are provided as a Source data file.

The C-terminal part of Gα that we analyzed with HDX-MS can be divided into two parts; one is the distal C-terminus (Fig. 3a, green-colored residues, ‘wavy hook’), and the other is the C-terminal helical region of α5 (Fig. 3a, black residues). In our previous study with the β_2_AR and Gs, we observed that the C-terminal part of Gα is the initial site that undergoes a change in hydroxyl radical foot printing upon interaction with the β_2_AR^19^. Moreover, the β_2_AR failed to induce GDP release from the wavy hook-truncated Gαs, which suggested that the wavy hook is the critical initial binding site during β_2_AR-Gs coupling^19^ (see below).

To test the role of the wavy hook in GPCR-Gi/o coupling, we also generated a Gαi3 mutant construct in which the last five residues were truncated (hereafter denoted Gi3_Δ5). Unlike β_2_AR-Gs coupling, both the M2R and the β_2_AR could induce GDP release from Gi3_Δ5 (Fig. 3b). Interestingly, β_2_AR-induced GDP release profile did not change upon C-terminal truncation, while M2R-induced GDP release was decreased so that the extent of this GDP release became similar between β_2_AR-Gi3, β_2_AR-Gi3_Δ5, and M2R-Gi3_Δ5 (Fig. 3b). These data suggest that the wavy hook of Gαi3 has a role in differentiating between primary and secondary coupling; it is likely that the engagement of the wavy hook of Gαi3 with a receptor is necessary for primary (i.e., strong) coupling, while failure in doing so (evident from HDX-MS studies of β_2_AR-Gi/o coupling or from GDP release analysis of M2-Gi3_Δ5 coupling) leads to secondary (i.e., weak) coupling.

### The role of the wavy hook for GPCR-Gi/o coupling selectivity

As Gi3_Δ5 could still couple with receptors (Fig. 3b), we hypothesized that truncation of the wavy hook of Gi3 results in loss of coupling selectivity and leads to promiscuous coupling with non-Gi/o-coupling receptors. To test this hypothesis, we analyzed the GDP release activity of the M1R on WT Gi3 and Gi3_Δ5. According to the IUPHAR/BPS Guide to Pharmacology and a previous report, the M1R primarily couples with Gq/11^24^ (Supplementary Fig. 1b), and we confirmed that the M1R does not couple with WT Gi3 (Fig. 3c). However, we clearly observed M1R-induced GDP release from Gi3_Δ5, although the coupling efficiency was low (approximately 20% GDP release) (Fig. 3c). These data support our hypothesis that truncation of the distal C-terminus of Gi3 induces promiscuous coupling with non-Gi/o-coupling receptors.

The potential mechanism of promiscuous coupling of Gi3_Δ5 with the M1R may be attributed to its higher intrinsic dynamics relative to WT Gi3. In other words, the wavy hook may assist in retaining Gi3 in the GDP-bound state. However, we did not detect any difference in basal GDP release between WT Gi3 and Gi3_Δ5 (Fig. 3c). To further investigate the basal GDP/GTP exchange tendency, we analyzed the uptake of BODIPY-conjugated GTPγS (BODIPY-GTPγS) into WT Gαi3 and Gαi3_Δ5. We used the α subunit of Gi3—without forming a heterotrimer with Gβγ—to facilitate GDP/GTP turnover (Fig. 3d, e). The BODIPY fluorescence increases when BODIPY-GTPγS is located within the nucleotide-binding pocket compared with when it is free in the buffer allowing the detection of GTPγS binding by measuring the BODIPY fluorescence^27,28^. BODIPY-GTPγS binding to WT Gαi3 and Gαi3_Δ5 occurred with similar kinetics (Fig. 3d), although the maximal binding was slightly higher in WT Gαi3 than that in Gαi3_Δ5 (Fig. 3e). These data suggest that the intrinsic GDP release activity of Gαi3_Δ5 is not higher than that of WT Gαi3, and therefore M1R-induced GDP release from Gi3_Δ5 is not due to increased intrinsic GDP release activity.

### Gi/o-induced HDX profile changes of M2R and β_2_AR

**Fig. 4:**
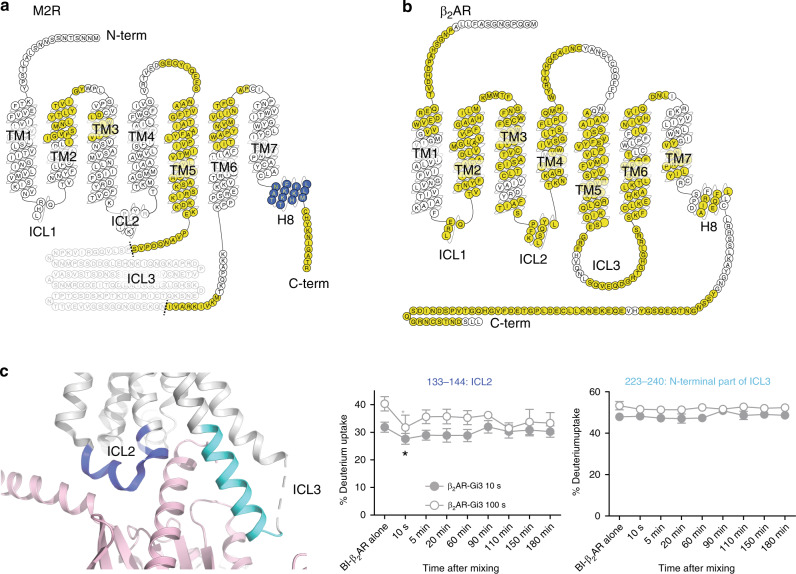
HDX profiles of the receptors during M2R-GoA and β_2_AR-GoA coupling. **a**, **b** HDX level changes of the M2R (**a**) and the β_2_AR (**b**) after 3 h of incubation with GoA. The changes in HDX are color-coded on the snake maps from GPCRdb (gpcrdb.org). Gray residues in M2R indicate truncated ICL3 for better expression and purification. White residues indicate regions without identified peptides; yellow residues indicate regions without HDX changes; and blue residues indicate regions with decreased HDX upon co-incubation with GoA. **c** Pulsed HDX-MS analysis of selected peptides of the β_2_AR during β_2_AR-Gi/o coupling. The selected peptides analyzed are color-coded on the X-ray crystal structure of β_2_AR (PDB 3SN6) and on the titles of the deuterium uptake graphs. Light pink color represents Gαs. Statistically significant changes during coupling were analyzed by repeated-measures ANOVA (rANOVA). To compare two different time points, a two-tailed paired Student’s *t*-test was performed, and *p* < 0.05 was considered as statistically significant. * Indicates the first time point that showed a statistical difference compared with pre-incubation. Error bars represent mean ± S.E.M of three independent experiments. Please note that the data is plotted using a non-linear/non-logarithmic scale.

To gain more insights into the structural changes that occur upon receptor-Gi/o coupling, we compared the HDX levels of the M2R and the β_2_AR before and after 3 h of co-incubation with GoA (Fig. 4a, b, and Supplementary Fig. 4b) and analyzed the time-resolved HDX changes in the M2R and the β_2_AR during M2R-Gi/o and β_2_AR-Gi/o coupling (Fig. 4c).

As discussed above, we have previously analyzed the HDX profile changes in the β_2_AR upon co-incubation with Gs, and observed decreased HDX at ICL2 and the N-terminal region of ICL3 of the β_2_AR (Supplementary Fig. 5a)^19^. The decreased HDX at ICL2 reflects helix formation and interaction of F139 with the hydrophobic pocket of Gαs (as shown in Supplementary Fig. 2a, blue dotted box), and the decreased HDX at the N-terminus of ICL3 reflects extended helix formation upon interaction with the C-terminal part of Gαs (as shown in Supplementary Fig. 2a, green square).

We could not identify peptides from ICL2 region of the M2R but could analyze the N-terminal region of ICL3. Unlike the β_2_AR-Gs complex, this region did not undergo HDX changes (Fig. 4a). This is consistent with the high-resolution structures of the M2R, where the N-terminal region of ICL3 does not form an extended helix upon interaction with GoA (Supplementary Fig. 4c). Helix 8 (H8) of the M2R showed decreased HDX (Fig. 4a and Supplementary Fig. 4b), which may be caused by the interaction between H8 and the C-terminal part of Gαi/o, as observed in the high-resolution structures of GPCR-Gi/o complexes^17^. Unfortunately, we could not obtain the pulsed HDX-MS data from H8, and therefore we could not correlate the time-course of conformational changes between the C-terminal part of Gαi/o and M2R H8.

For the β_2_AR-Gi/o complex, when we compared the HDX profiles before and after 3 h of co-incubation of the β_2_AR with GoA, HDX levels on the β_2_AR were not altered at any of the analyzed regions (Fig. 4b). A time-resolved HDX-MS study also showed that the N-terminal region of ICL3 of the β_2_AR does not undergo decreased HDX at any of the tested timepoints during coupling with Gi3 (Fig. 4c, peptides 223‒240). A lack of HDX change at the N-terminus of ICL3 suggests that the β_2_AR fails to form extended helices at the N-terminal region of ICL3 when coupled with Gi/o. This observation is consistent with the lack of HDX change at the C-terminal part of GαoA or Gαi3 (Fig. 1d, Fig. 2, and Supplementary Fig. 5b), which strengthens the hypothesis that the C-terminus of GαoA or Gαi3 is not deeply inserted into the β_2_AR core.

However, time-resolved analysis of HDX profile change showed that the HDX level at ICL2 underwent a transient decrease (within 10 s of co-incubation) upon co-incubation with Gi3 (Fig. 4c, peptides 133‒144). After 10 s, the HDX level was not statistically different either from the 10 s time point or from the point of β_2_AR alone. These results suggest that ICL2 engages with Gi3 during an early event, but that the interaction may not be stable.

### Importance of ICL2 in primary and secondary Gi/o coupling

**Fig. 5:**
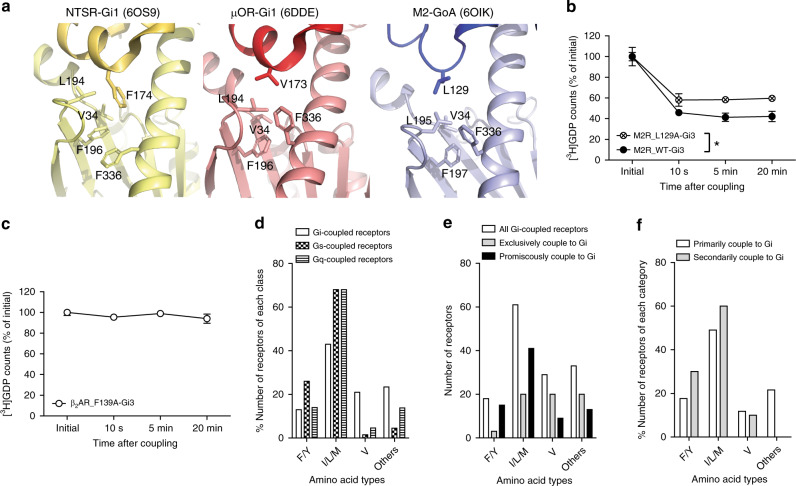
The role of residue 34.51 within ICL2 in M2R-Gi3 and β_2_AR-Gi3 coupling. **a** The structures of ICL2 and hydrophobic pocket of neurotensin receptor-Gi1 complex (left, PDB 6OS9), mu-opioid receptor-Gi1 complex (middle, PDB 6DDE), and M2R-GoA complex (right, PDB 6OIK). Receptors are shown in dark color, and G proteins are shown in light color. **b** GDP release profiles of M2R-Gi3 and M2R_L129A-Gi3. **c** GDP release profiles of β_2_AR_F139A-Gi3. For (**b**) and (**c**), * indicates statistical differences between groups as analyzed by a two-tailed Student’s *t*-test (*p* < 0.05). Error bars represent mean ± S.E.M of three independent experiments. Please note that the data is plotted using a non-linear/non-logarithmic scale. **d** Amino acid type analysis at residue 34.51 of type A GPCRs with known coupling G proteins. **e** Amino acid type analysis at residue 34.51 of type A Gi/o-coupled receptors subcategorized into exclusive or promiscuous coupling. **f** Amino acid type analysis at residue 34.51 of promiscuously Gi/o-coupled receptors, which are subcategorized into primary and secondary coupling. Source data are provided as a Source data file.

Our previous studies suggested that for β_2_AR-Gs coupling, the interaction of a bulky hydrophobic residue, F139 at ICL2, (residue 34.51, based on GPCRdb numbering scheme) with the hydrophobic pocket formed by H41, V217, F219, and F376 (Supplementary Fig. 2a) is the critical step to induce GDP release^19,29^. For example, mutation of Phe139 to Ala in the β_2_AR (hereafter denoted β_2_AR_F139A) abolished β_2_AR-induced GDP release from Gs, although this construct could still interact with Gs^19^. On the other hands, the currently available high-resolution structures of GPCR-Gi/o complexes show that hydrophobic residues at 34.51 form weak hydrophobic interactions with the hydrophobic pocket formed by V34, L194/L195, F196/F197, and F336 of the Gαi/o families (Fig. 5a).

To test the role of the interaction of residue 34.51 in primary and secondary GPCR-Gi coupling, we generated mutant constructs in which the bulky hydrophobic residue at 34.51 was replaced with Ala (M2R_L129A and β_2_AR_F139A). M2R_L129A could still induce GDP release from Gi3, albeit to a lesser degree than WT M2R (Fig. 5b). This result is consistent with the previous report in which M2R_L129A induced GDP/GTP turnover, although the degree was reduced to 50% relative to that in WT M2R^16^. Unlike M2R_L129A-Gi3 coupling, β_2_AR_F139A failed to release GDP from Gi3 (Fig. 5c), which suggests that the bulky hydrophobic residue 34.51 at ICL2 is critical for β_2_AR-Gi3 coupling.

To gain more insights into the role of residue 34.51 in GPCR-Gi/o coupling, we analyzed the amino acid residue at position 34.51 in class A GPCRs with known coupling G proteins (Supplementary Fig. 1 and Fig. 5d–f). Among the Gs-coupled receptors, 26% contain Phe or Tyr, and 68% contain large hydrophobic residues such as Ile, Leu, or Met (Fig. 5d). Thus, a majority (94%) of Gs-coupled receptors comprise of very large/large hydrophobic or aromatic ring-containing amino acid residues at position 34.51. Similarly, a majority (82%) of Gq/11-coupled receptors contain very large/large hydrophobic or aromatic ring-containing amino acid residues at position 34.51 (Fig. 5d). In contrast, the proportion of very large/large hydrophobic or aromatic ring-containing amino acid residues at position 34.51 decreases in Gi/o-coupled receptors (56%), while the proportion of medium or small hydrophobic amino acids at position 34.51 increases in Gi/o-coupled receptors (25.3%) (Fig. 5d). Moreover, 17.7% of Gi/o-coupled receptors contain non-hydrophobic residues (His, Pro, Ser, Thr, Arg, Lys, Glu, and Gln) whereas only 4.5% of Gs-coupled receptors and 13.1% of Gq/11-coupled receptors contain these residues (Fig. 5d). These sequence analyses imply that the bulky hydrophobic residue at 34.51 is important for Gs or Gq/11 coupling, but this may not be necessarily true for Gi/o coupling.

We further analyzed amino acids at position 34.51 of all class A Gi/o-coupled receptors (Fig. 5e). Comparison of the exclusively-Gi/o-coupled and promiscuously-Gi/o-coupled receptors revealed a broad range of amino acid residues in the exclusively-Gi/o-coupled receptors contain, whereas the promiscuously-Gi/o-coupled receptors contain mostly very large/large hydrophobic or aromatic ring-containing amino acid residues (56 receptors out of 78 promiscuously-Gi/o-coupled receptors) (Fig. 5e). When we further subcategorized the promiscuously-Gi/o-coupled receptors into primarily-Gi/o-coupled receptors and secondarily-Gi/o-coupled receptors, we found that all the receptors that secondarily couple to Gi/o contain Phe/Tyr, Ile/Leu/Met, or Val and do not contain other amino acids while the receptors that primarily couple to Gi/o contain these amino acids as well as other residues such as Ala, His, Pro, Ser/Thr, Arg/Lys, and Gln (Fig. 5f). These findings imply that for the secondary GPCR-Gi/o interaction, the bulky hydrophobic residue at ICL2 may be important, which is consistent with the observation that β_2_AR_F139A failed to release GDP from Gi3 (Fig. 5c).

Taken together, we suggest that the interaction of residue 34.51 with the hydrophobic pocket within the Gα subunit is not critical for primary GPCR-Gi/o coupling, but it is important for secondary GPCR-Gi/o coupling.

## Discussion

Previous studies have suggested that a single receptor differentially activates different G proteins to varying degrees and/or with different kinetics, which results in complex signal transduction^6,9,30^. This fine-tuning of GPCR-G protein coupling is important for the precise regulation of cellular functions. However, only few studies have suggested the mechanism underlying differential coupling of promiscuous receptors; for example, the availability of G proteins limits the GPCR-G protein coupling selectivity^4,7^, and different ligand types or ligand concentrations differentially regulate the promiscuity of GPCR-G protein coupling^31,32^.

**Fig. 6:**
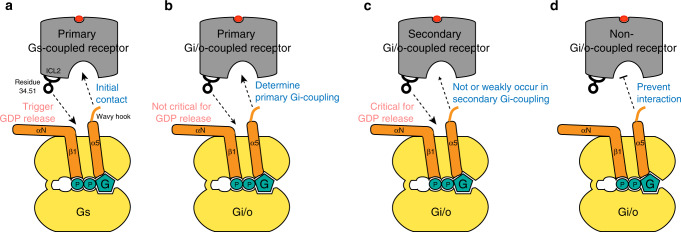
Summary of Gs and Gi/o coupling mechanisms. **a** The structural mechanism of primary Gs-coupling based on the study by Du et al.^19^ is illustrated. Wavy hook is the initial contact site with a receptor followed by interaction of residue 34.51 at ICL2 with Gαs, which triggers GDP release. **b**, **c** The structural mechanism of primary and secondary Gi/o-coupling is illustrated. Interaction of the wavy hook with the receptor determines primary and secondary Gi/o coupling. Interaction of residue 34.51 at ICL2 with Gαi/o is critical for secondary Gi/o-coupling but not for primary Gi/o-coupling. **d** The wavy hook of Gαi/o prevents coupling with non-Gi/o-coupled receptors.

The current study presents the conformational factors that differentiate between primary and secondary Gi/o-coupling. We found that one of the key structural factors is the engagement of the wavy hook of Gαi/o with the receptor (Fig. 6b vs. Fig. 6c). Failure of strong engagement of the wavy hook with the receptor leads to secondary Gi/o coupling (i.e., β_2_AR-Gi/o, β_2_AR-Gi3_Δ5, or M2R-Gi3_Δ5).

These results are surprising because the engagement of the distal C-terminus of Gα with receptors has been considered to be critical for GPCR-G protein interaction and coupling selectivity^19,20,33–39^. In our previous study using the β_2_AR and Gs, truncation of the wavy hook resulted in the complete loss of β_2_AR-induced GDP release, and we suggested that the wavy hook is the initial binding site^19^ (Fig. 6a). However, the present data indicate that the M2R and the β_2_AR could still couple to the ‘wavy hook-truncated’ Gαi3 (Fig. 3b), and moreover, the M1R, a non-Gi/o-coupled receptor, can induce GDP release from Gi3_Δ5 but not from WT Gi3 (Fig. 3c). It is tempting to suggest that the wavy hook of Gi3 facilitates interaction with primary Gi/o-coupled receptors but inhibits interaction with non-Gi/o-coupled receptors (Fig. 6d). The detailed molecular mechanism of the role of the wavy hook in differentiating primary vs. secondary Gi/o-coupling and preventing interaction with the non-Gi/o-coupled receptors needs further investigation using systematic mutagenesis or swap mutation of the wavy hook.

Different coupling modes between primary and secondary Gi/o-coupling were also found at receptor residue 34.51 (Fig. 6b vs. Fig. 6c) as the bulky hydrophobic residue at 34.51 is critical for β_2_AR-Gi3 coupling but not for M2R-Gi3 coupling (Fig. 5b, c). Previous studies reported that residue 34.51 within ICL2 of the receptor is critical for receptor-G protein coupling^19,40^. Interestingly, the importance of residue 34.51 has been mostly observed for GPCR-Gs interaction^19,40^ but not for GPCR-Gi/o interaction (Supplementary Fig. 2a vs. Fig. 5a). The analysis of the amino acid type at residue 34.51 supports the hypothesis that the bulky hydrophobic residue at 34.51 is important for primary GPCR-Gs or GPCR-Gq/11 coupling, but may not be critical for primary GPCR-Gi/o coupling (Fig. 5). Interestingly, all the receptors that secondarily couple to Gi/o contain large hydrophobic amino acids at residue 34.51 (Fig. 5f) suggesting that such amino acids at position 34.51 may be necessary for secondary GPCR-Gi/o coupling.

The current study also suggests that primary Gi/o coupling might follow certain different structural mechanisms compared with primary Gs coupling (Fig. 6a vs Fig. 6b). First, αN showed increased HDX in β_2_AR-Gs coupling and decreased HDX in M2R-Gi3/GoA coupling (Fig. 2 and Supplementary Fig. 5). Second, the β_2_AR failed to induce GDP release from Gs_Δ5^19^, but the M2R still induced GDP release from Gi3_Δ5 (Fig. 3b). Third, β_2_AR_F139A failed to induce GDP release from Gs^19^, but M2R_L129A still induced GDP release from Gi3 (Fig. 5b). These discrepancies may provide clues to understand GPCR-G protein selectivity, which needs further investigation.

In conclusion, we propose the potential conformational mechanism differentiating primary and secondary Gi/o-coupling and compared the conformational mechanism of primary Gi/o-coupling with that of primary Gs-coupling. The findings raise questions about the detailed functional mechanism of the wavy hook in facilitating primary Gi/o coupling and preventing non-Gi/o coupling. Moreover, the critical step for GDP release during primary GPCR-Gi/o coupling is remained to be elucidated because the interaction of the residue 34.51 was not critical for GPCR-Gi/o coupling as suggested in GPCR-Gs coupling.

## Methods

### Expression and purification of Gi/o

Following protocol is for expression and purification of samples used for Figs. 1, 2, 4, and 5. Human GαoA or Gαi3 was cloned into pFastBac1 vector; Gβ1 with 3C protease-cleavable 6xHis-tag and Gγ2 were cloned into pFastBac_Dual vector. The G proteins were expressed in High Five insect cells (Expression Systems, 94–001F) using Bac-to-Bac system. Cell cultures were grown at 27 °C to a density of 3 × 10^6^ cells mL^−1^ and then infected with Gαo or Gαi3 and Gβ1γ2 baculovirus (10–20 mL L^−1^ and 1–2 mL L^−1^ respectively). After 48 h incubation, the infected cells were harvested by centrifugation and stored at −80 °C until use.Cell pellets were resuspended in 100 mL lysis buffer (10 mM Tris, pH 7.5, 0.1 mM MgCl_2_, 5 mM β-mercaptoethanol (β-ME), 10 μM GDP, 2.5 μg mL^−1^ leupeptin, and 160 μg mL^−1^ benzamidine) per liter of culture volume and stirred at room temperature for 15 min. Cell membranes were then spun down and resuspended in 100 mL solubilization buffer (20 mM HEPEs, pH 7.5, 100 mM NaCl, 1% sodium cholate, 0.05% DDM, 5 mM MgCl_2_, 2 μL CIP, 5 mM β-ME, 15 mM imidazole, 10 μM GDP, 2.5 μg mL^−1^ leupeptin, and 160 μg mL^−1^ benzamidine) per liter of culture volume using a Dounce homogenizer. The sample were stirred at 4 °C for 1 h, and then centrifuged for 20 min to remove insoluble debris. Nickel-NTA resin (2 mL L^−1^ cell culture) pre-equilibrated in solubilization buffer was added to the supernatant and shaken for 2 h at 4 °C. After incubation, the Ni-NTA resin was spun down, poured into a glass column, and washed with 50 mL solubilization buffer. The heterotrimeric GoA or Gi3 was then gradually exchanged into E2 buffer (20 mM HEPEs pH 7.5, 50 mM NaCl, 0.1 % DDM, 1 mM MgCl_2_, 5 mM β-ME, 10 μM GDP, 2.5 μg mL^−1^ leupeptin, and 160 μg mL^−1^ benzamidine). The protein was then eluted with E2 buffer supplemented with 250 mM imidazole. The protein was then dephosphorylated by treating with 5 μL lamda phosphatase (supplemented with 1 mM MnCl_2_ for activity), 1 μL CIP, and 1 μL Antarctic phosphatase, then incubated at 4 °C overnight. The 6xHis-tag was removed using 3C protease. Cleaved GoA or Gi3 was purified by an additional negative Ni-NTA purification step. The Ni-NTA chromatography purified GoA or Gi3 was further purified with MonoQ column (GE Healthcare). The peak fractions of MonoQ column were collected and concentrated using a 50 kDa molecular weight cutoff Millipore concentrator. The concentrated heterotrimeric GoA or Gi3 was aliquoted, flash frozen in liquid nitrogen and frozen at −80 °C before use.

### Expression and purification of WT and mutant Gαi3_Δ5

Following protocol is for expression and purification of samples used for Fig. 3. The recombinant Gαi/o protein containing N-terminal His-tag and TEV cleavage site was constructed in pET21a. Gαi3_Δ5 mutant was generated by site-directed mutagenesis using PCR. The primers used for mutagenesis are listed in Supplementary Table 1. Gαi3 and Gαi3_Δ5 were transformed into *Escherichia coli* LOBSTR (Kerafast, EC1002) and GαoA was transformed into *Escherichia coli* BL21-DE3 (iNtRON, ITY-YE207). Cells were grown in Terrific Broth in the presence of antibiotic at 37 °C until OD_600_ reached 0.6–0.8. Protein expression was induced by 0.03 mM IPTG and cells were further incubated at 16 °C for 24 h. For protein purification, the cell pellets were harvested and resuspended in lysis buffer (20 mM HEPES pH 7.4, 300 mM NaCl, 2 mM MgCl_2_, 20 µM GDP, 1:1000 protease inhibitor cocktail, 2.5 µg ml^−1^ leupeptin, 10 µg ml^−1^ benzamidine, 100 µM TCEP, and 10% glycerol) in three folds of cell pellet volume and incubated with 5 mg mL^−1^ lysozyme for 30 min at room temperature. The lysate was then incubated with 10 µg mL^−1^ DNaseI for another 30 min. The supernatant was collected by centrifugation at 20,000 × *g* for 30 min at 4 °C, supplemented with 20 mM imidazole, and loaded onto Ni-NTA column equilibrated with lysis buffer containing 20 mM imidazole. The Ni-NTA resin was washed with wash buffer (20 mM HEPES pH 7.4, 300 mM NaCl, 2 mM MgCl_2_, 20 µM GDP, 1:1000 protease inhibitor cocktail, 2.5 µg mL^−1^ leupeptin, 10 µg mL^−1^ benzamidine, 100 µM TCEP, and 20 mM imidazole). The proteins were eluted with elution buffer (20 mM HEPES pH 7.4, 300 mM NaCl, 2 mM MgCl2, 20 µM GDP, 1:1000 protease inhibitor cocktail, 2.5 µg mL^−1^ leupeptin, 10 µg mL^−1^ benzamidine, 100 µM TCEP, and 250 mM imidazole). Proteins were further purified using a Superdex-200 (10/300) column with ÄKTA FPLC. The protein fractions were collected by monitoring the absorbance at 280 nm. Proteins were then concentrated, supplemented with 20% glycerol, and stored at −80 °C until further use.

### Expression and purification of the M2R and the M1R

Human M2R and M1R genes, containing N-terminal FLAG-tag and C-terminal His-tag, were subcloned into pFastBac1 vector. The L129A mutation of M2R was generated by Quick-Change mutagenesis and confirmed by DNA sequencing. The primers used for mutagenesis are listed in Supplementary Table 1. All M2R and M1R constructs used in this study were expressed in Sf9 insect cells (Expression Systems, 94–002F) using the Bac-to-Bac baculovirus system. Sf9 cells were grown in the ESF 921 medium and were infected with recombinant baculovirus at a density of 4 × 10^6^ cells mL^−1^, in the presence of 10 μM atropine. The cells were harvested after 48 h of infection at 27 °C. Cell pellets were lysed using a lysis buffer (10 mM Tris pH 7.5, 1 mM EDTA, 10 μM atropine, 2.5 μg mL^−1^ leupeptin, and 160 μg mL^−1^ benzamidine). Cell membranes were then spun down and solubilized with a buffer containing 20 mM HEPES (pH 7.5), 750 mM NaCl, 1% DDM, 0.2% sodium cholate, 0.03% CHS, 10 μM atropine, 2.5 μg mL^−1^ leupeptin, 160 μg mL^−1^ benzamidine, and 30% glycerol. The solubilized receptor was then purified through Ni-NTA chromatography and eluted with a buffer containing 20 mM HEPES (pH 7.5), 750 mM NaCl, 0.1% DDM, 0.02% sodium cholate, 0.03% CHS, 10 μM atropine, and 30% glycerol and supplemented with 250 mM imidazole. The Ni-NTA purified receptor was then loaded onto an anti-FLAG column with M1 affinity resin and washed extensively with a buffer containing 20 mM HEPES (pH 7.5), 750 mM NaCl, 0.1% DDM, 0.02% sodium cholate, 0.003% CHS, and 10 μM iperoxo and supplemented with 2 mM CaCl_2_. Thereafter, it was eluted with the same buffer supplemented with 0.2 mg mL^−1^ of FLAG peptide and 5 mM of EDTA. The anti-FLAG-chromatography-purified receptor was finally purified by size exclusion chromatography against a buffer containing 20 mM HEPES (pH 7.5), 100 mM NaCl, 0.1% DDM, 0.003% CHS, and 10 μM iperoxo. The monodisperse peak fractions were concentrated, flash frozen, and stored at −80 °C until further use.

### Expression and purification of the β_2_AR

The β_2_AR was expressed in Sf9 insect cells (Expression Systems, 94–002F) using the BestBac expression system. The F139A mutation was generated by Quick-Change mutagenesis and confirmed by DNA sequencing. The primers used for mutagenesis are listed in Supplementary Table 1. Proteins were expressed by infecting sf9 cells at 4 × 10^6^ cells mL^−1^ with second-passage baculovirus using 20 mL L^−1^ of cell culture supplemented with 2 µM alprenolol. The cells were harvested after 48 h incubation at 27 °C. Cell pellets were resuspended in lysis buffer (20 mM HEPES pH 7.5, 5 mM EDTA, 1 µM alprenolol, 2.5 µg mL^−1^ leupeptin, and 160 µg mL^−1^ benzamidine) at 10 mL g^−1^ of cell pellet and stirred for 15 min. The collected cell membrane was then homogenized by a Douncer device with the sample in solubilization buffer (20 mM HEPES, pH 7.5, 100 mM NaCl, 1% DDM, 1 µM alprenolol, 2.5 µg mL^−1^ leupeptin, and 160 µg mL^−1^ benzamidine) for 1 h at room temperature to extract the receptor. After adding 2 mM CaCl_2_, the supernatant was collected by centrifugation at 18,000 × *g* for 30 min and loaded onto anti-FLAG column with M1-antibody resin. The column was thoroughly washed with HMS-CHS buffer (20 mM HEPES pH 7.5, 350 mM NaCl, 0.1% DDM, and 0.01% cholesterol hemisuccinate) supplemented with 2 mM CaCl_2._ The receptor was then eluted with HMS-CHS buffer supplemented with 5 mM EDTA and 200 µg mL^−1^ FLAG peptide. The eluted protein was kept frozen and thawed immediately prior to use. The thawed receptor was further purified via alprenolol-Sepharose affinity chromatography using HMS-CHS buffer with 300 µM alprenolol as the elution buffer. The eluted receptors were once again loaded onto anti-FLAG column and washed with HMS-CHS buffer to achieve unliganded receptors by removing alprenolol. The bound receptor was then eluted with HMS-CHS buffer with 5 mM EDTA, 200 µg ml^−1^ FLAG peptide and 10 µM BI-167107. The functional receptors were further purified by size exclusion chromatography using a Superdex-200 column in HLS-CHS buffer 20 mM HEPES pH 7.5, 150 mM NaCl, 0.1% DDM, 0.01% CHS, 2 µM BI-167107. The receptors were concentrated, flash frozen in liquid nitrogen, and stored at −80 °C until use.

### GPCR-Gi/o complex formation and HDX-MS

To form GPCR-Gi/o complex, Gi/o (65 µM) was mixed with 1.15-fold molar excess of iperoxo-bound M2R or BI-167107-bound β_2_AR at room temperature. Apyrase (200 mU mL^−1^) was added after 90 min of incubation to hydrolyze GDP, and to generate a stable complex. For continuous labeling deuterium exchange, 5 µL of complex, agonist bound receptor or GDP-bound Gi/o was mixed with 25 µL of D_2_O buffer (20 mM HEPES (pD 7.4), 100 mM NaCl, 100 mM TCEP, and 0.1% DDM supplemented with 5 µM agonist, 20 µM GDP, or both for receptor alone, G protein alone, or complex, respectively) and incubated for 10, 100, 1000, and 10,000 s at room temperature. For pulse-labeling deuterium exchange, GPCR and Gi/o were mixed at room temperature as described above, and 5 µL aliquots were collected at the indicated time points (before mixing, 10 s, 5 min, 20 min, 60 min, 90 min, 110 min, 150 min, and 180 min), mixed with 25 µL of D_2_O buffer, and incubated for 10 s or 100 s at room temperature. The deuterated samples were quenched using 30 µL of ice-cold quench buffer (0.1 M NaH_2_PO_4_ and 20 mM TCEP (pH 2.01)), snap-frozen on dry ice, and stored at −80 °C. Non-deuterated samples were prepared by mixing 5 µL of protein sample with 25 µL of their respective H_2_O buffers, followed by quenching and freezing, as described above.

The quenched samples were digested and isolated using the HDX-UPLC-ESI-MS system (Waters, Milford, MA, USA). Briefly the quenched samples were thawed and immediately injected to an immobilized pepsin column (2.1 × 30 mm) (Life Technologies, Carlsbad, CA, USA) at a flow rate of 100 µL min^−1^ in 0.05% formic acid in H_2_O at 12 °C. The peptic peptides were then collected on a C18 VanGuard trap column (1.7 µm × 30 mm) (Waters) for desalting with 0.05% formic in H_2_O and subsequently separated by ultra-pressure liquid chromatography using an Acquity UPLC C18 column (1.7 µm, 1.0 × 100 mm) (Waters) at a flow rate of 40 µL min^−1^ with an acetonitrile gradient starting from 8 to 85% over 8.5 min using two pumps. The mobile phase A was 0.1% formic acid in H_2_O and the mobile phase B was 0.1% formic acid in acetonitrile. Buffers were adjusted to pH 2.5 and the system was maintained at 0.5 °C (except pepsin digestion was performed at 12 °C) to minimize the back-exchange of deuterium to hydrogen. Mass spectra were analyzed by Xevo G2 quadrupole-time of flight (Q-TOF) equipped with a standard electrospray ionization (ESI) source in MS^E^ mode (Waters) with positive ion mode. The capillary, cone, and extraction cone voltages were set to 3 kV, 40 V, and 4 V, respectively. Source and desolvation temperatures were set to 120 °C and 350 °C, respectively. Trap and transfer collision energies were set to 6 V, and the trap gas flow was set to 0.3 mL min^−1^. The mass spectrometer was calibrated with sodium iodide solution (2 µg µL^−1^). [Glu1]-Fibrinopeptide B solution (200 fg µL^−1^) in MeOH:water (50:50 (v/v) + 1% acetic acid) was utilized for the lock-mass correction and the ions at mass-to-charge ration (*m/z*) 785.8427 were monitored at scan time 0.1 s with a mass window of ±0.5 Da. The reference internal calibrant was introduced at a flow rate of 20 µL min^−1^ using the lock mass sprayer and the acquired spectra were automatically corrected using the lock-mass. Two independent interleaved acquisitions were automatically created: the first function, typically set at 4 eV, collected low energy or unfragmented data while the second function collected high energy or fragmented data typically obtained by using a collision ramp from 30–55 eV. Ar gas was used for collision induced dissociation (CID). Mass spectral were acquired in the range of m/z 100–2000 for 10 min. ProteinLynx Global Server 2.4 (Waters) was utilized to identify peptic peptides from the non-deuterated samples with variable methionine oxidation modification and a peptide score of 6. DynamX 2.0 software (Waters) was used to determine the level of deuterium uptake for each peptide by measuring the centroid of isotopic distribution. The average back-exchange in our system was ~30‒40%, but we did not correct for back exchange because the proteins aggregate in fully deuterated samples. All the data were derived from at least three independent experiments. The detailed HDX-MS results are summarized in the supplementary data, which were generated according to Masson et al.’s recommendation^41^.

### GDP release assay

Purified Gα subunit (200 nM) of each G protein (GoA or Gi3) was mixed with 50 nM of [^3^H]GDP for 1 h at room temperature, in a buffer containing 20 mM HEPES (pH 7.5), 100 mM NaCl, 0.1% DDM, 100 μM TCEP, and 2 μM GDP. Thereafter, 2 μM of purified Gβγ was added. After 10 min of incubation, 5 μM of BI-167107-bound β_2_AR, iperoxo-bound M2R, or the corresponding DDM buffer, of similar volume, was further added to initiate GDP release, in the presence of 1 μM GDP. The reaction mixture was aliquoted at indicated time points, and immediately loaded onto calibrated G-50 columns. The flow through was collected with 1.1 mL of buffer (20 mM HEPES (pH 7.5), 100 mM NaCl, and 0.1% DDM), and GoA- or Gi3-bound [^3^H]GDP was measured using a scintillation counter (Beckman Coulter, Brea, CA, USA), after adding 15 mL of scintillation fluid. The initial sample represents [^3^H]GDP binding capacity of GoA or Gi3, before initiation of GDP release.

### BODIPY-GTPγS assay

Nucleotide binding to GαoA and Gαi3 were determined by measuring change in fluorescence intensity of BODIPY-FL-GTPγS (Thermo Fisher Scientific, Waltham, MA, USA) in an imaging buffer comprised of 20 mM Tris-HCl (pH 8.0), 1 mM EDTA, 10 mM MgCl_2_, and 100 µM TCEP. The fluorophore was excited at 485 nm (bandwidth 14 nm) and the emission spectrum was recorded at 535 nm (bandwidth 25 nm) using TriStar2 S LB 942 Multimode Microplate Reader (Berthold, Germany). Baseline in absence of protein samples was determined by measuring fluorescence intensity of imaging buffer with or without 50 nM BODIPY-FL-GTPγS for 120 s. GαoA and Gαi3 prepared in 20 mM HEPES (pH 7.4), 100 mM NaCl, 2 mM MgCl_2_, 100 µM TCEP, and 10 µM GDP were mixed with imaging buffer with or without 50 nM BODIPY-FL-GTPγS in 1:10 dilution (1.5 µM final GαoA and Gαi3 concentration). The changes in fluorescence were measured for 60 min. Data points were collected every 10 s using a 96-well black plate. All steps were carried out at room temperature. The spectra were corrected by measurements in absence of BODIPY-FL-GTPγS and normalized by setting the peak fluorescence of BODIPY-FL-GTPγS in presence of protein as 100.

### Statistics and reproducibility

Results are representatives of at least three independent experiments and are expressed as mean ± S.E.M. Statistical analysis was performed using GraphPad Prism software (San Diego, CA, USA). Statistical significance of time-dependent changes was determined by repeated-measures one-way ANOVA (rANOVA) at an α level = 0.01 followed by Turkey’s multiple comparison test; change in a time series as a whole was considered significant when the F statistic was >1. The significance of differences between two different time points within series or two groups was determined by paired or unpaired two-tailed Student’s *t*-test. The significance of differences between more than two groups was determined by one-way ANOVA. Analysis was considered significant at *p* < 0.05.

### Reporting summary

Further information on research design is available in the Nature Research Reporting Summary linked to this article.

## Supplementary information

Supplementary Information

Description of Additional Supplementary Information

Supplementary Data 1

Reporting Summary

## Data Availability

Data supporting the findings of this manuscript are available from the corresponding authors upon reasonable request. A reporting summary for this Article is available as a Supplementary Information file. HDX-MS data have been deposited to ProteomeXchange Consortium^42^ via PRIDE^43^ partner repository with the set identifier PXD019367. The source data underlying Figs. 1b, 3b–e, and 5b–f are provided as a Source Data file. Data for sequence analysis in Fig. 5d–f are available from GPCR database (gpcrdb.org).

## Source data

Source Data
