# Supplementary material for: Structural mechanism underlying primary and secondary coupling between GPCRs and the Gi/o family
Source: Nat Commun. 2020 Jun 22;11:3160. doi: 10.1038/s41467-020-16975-2 (PMC7308389; doi:10.1038/s41467-020-16975-2)
Supplement: Supplementary file 1 — Supplementary Information [file 41467_2020_16975_MOESM1_ESM.pdf]

Supplementary Information

Structural mechanism underlying primary and secondary coupling between GPCRs and the Gi/o family

Kim et al.

<u>Brief description of what this file includes:</u>

Supplementary Fig 1. Database of G protein coupling of type A GPCRs.

Supplementary Fig 2. Important binding surface highlighted in high-resolution GPCR-Gs and GPCR-Gi/o complexes.

Supplementary Fig 3. HDX-MS profiles of GDP-bound Gαs and β_2AR-bound nucleotide-free Gαs from previous report.

Supplementary Fig 4. Deuterium uptake plots of peptides from blue- or red-colored regions in figures 1c,1d, and 4a.

Supplementary Fig 5. Time-resolved HDX-MS profiles during β_2AR-Gs, M2R-Gi3, and β_2AR-Gi3 coupling

Supplementary Table 1. Primers used for mutagenesis

<u>Other supplementary information of this manuscript includes:</u>

Reporting summary

Supplementary Data file. HDX-MS data summary and HDX-MS deuterium uptake levels

(Above data are deposited to ProteomeXchange Consortium via PRIDE partner repository with the set identifier PXD019367)

Source Data file. The source data underlying Fig. 1b, 3b-e, and 5b-f are provided.

a

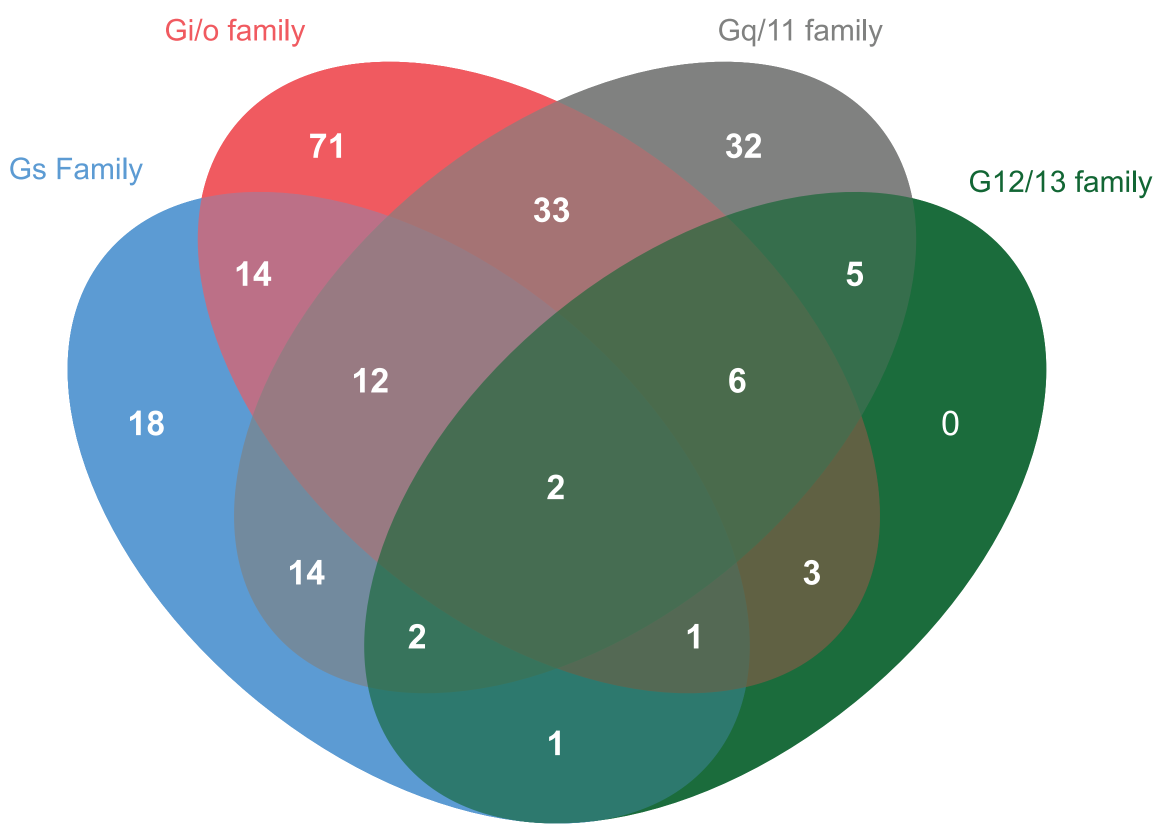

b

	β2AR	M2R	M1R
Primary coupling	Gs	Gi/o	Gq/11
Secondary coupling	Gi/o	Gs, Gq/11	-

Supplementary Fig. 1. a The number of type A GPCRs coupled to Gs, Gi/o, Gq/11, and G12/13 family proteins. The graph is derived from GPCRdb (gpcrdb.org). **b** The primary and secondary coupling G proteins for the β2AR, M2R, and M1R.

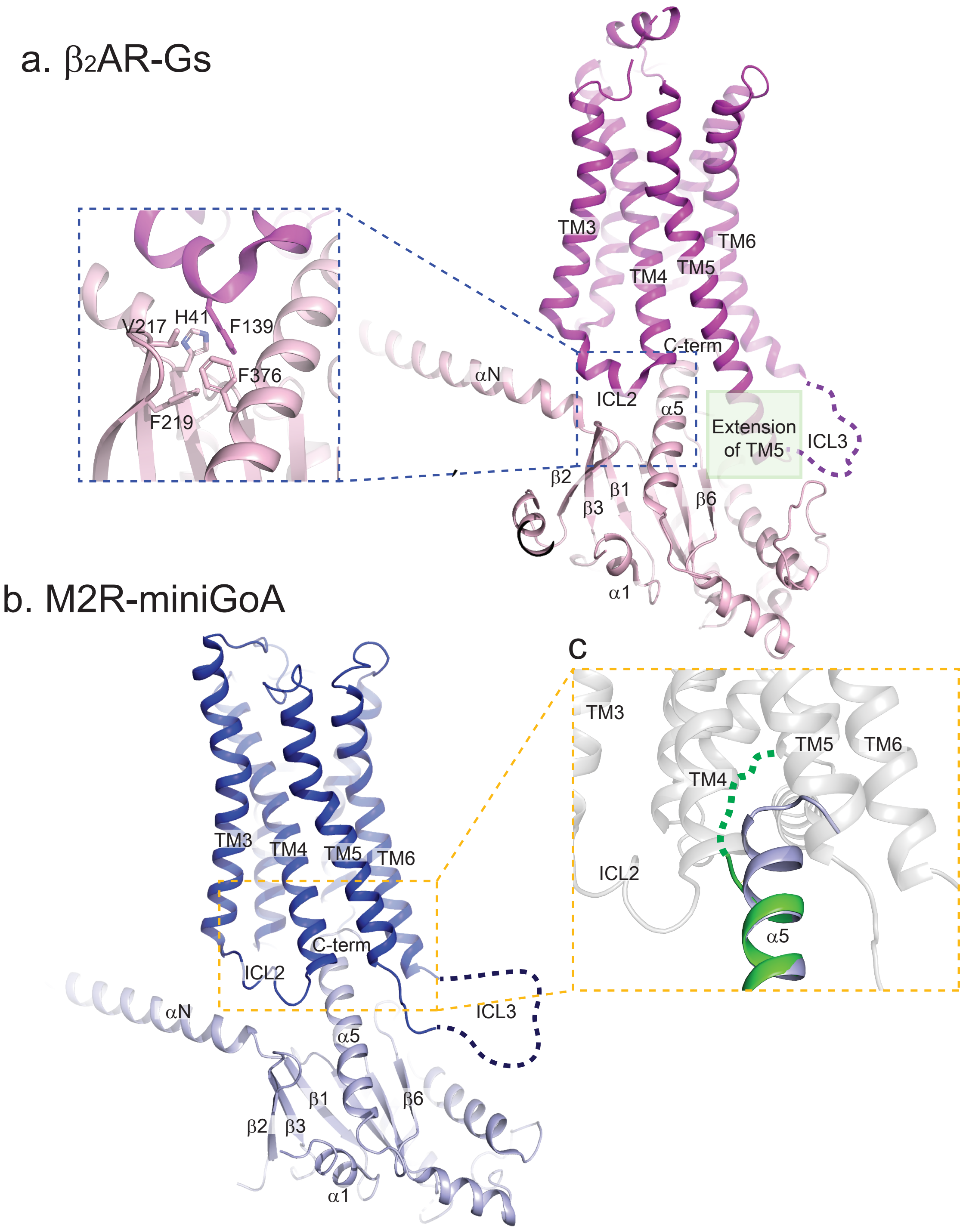

Supplementary Fig. 2. High-resolution structures of GPCR-Gs and GPCR-Gi complexes. **a** The X-ray crystal structure of β2AR-Gs (PDB 3SN6). The β2AR is in purple and Gs in light pink. Interaction of F139 within β2AR ICL2 with the hydrophobic pocket of Gαs is shown in the dotted blue box. The extended TM5 α-helix at the N-terminus of ICL3 is indicated with green-colored box. The α-helical domain of Gαs, Gβγ, T4 lysozyme fused to the N-terminus of the β2AR and nanobody are omitted for simplicity. Unstructured ICL3 is shown by dotted line. **b** The cryoEM structure of M2R-GoA (PDB 6OIK). The M2R is in dark blue and GαoA in light blue. Unstructured ICL3 is shown by dotted line. **c** The C-terminal part of Gαi/o is enlarged. GαoA complexed with the M2R is in light blue (PDB 6OIK) and Gαi1 in GDP-bound form is in green (PDB 1GP2). The wavy hook is not revealed in the GDP-bound strcucture and thus shown as a green dotted line. The M2R is shown in transparent grey.

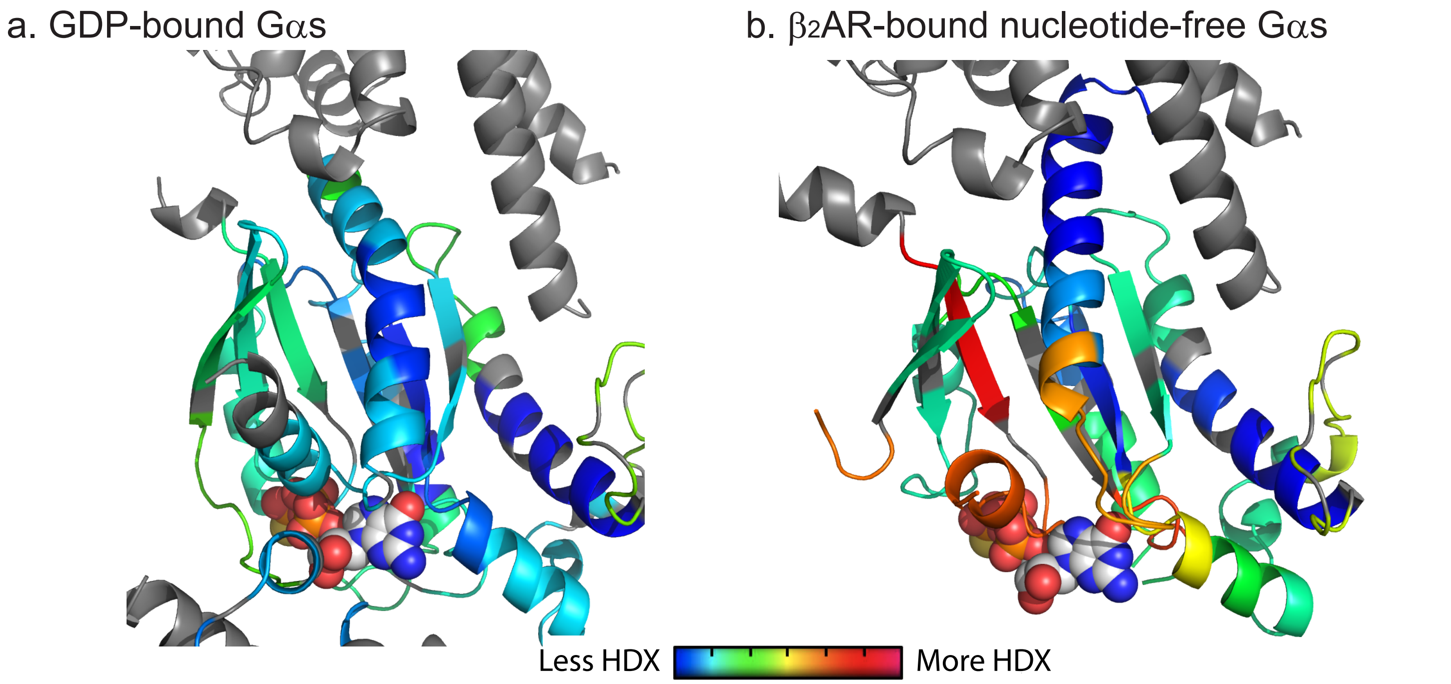

Supplementary Fig. 3. HDX-MS analysis of GDP-bound Gαs (**a**) and β2AR-bound nucleotide-free Gαs (**b**). The figure was re-produced based on the data previously published by Chung *et al.* (25). **a**. The HDX levels of GDP-bound Gαs were color-coded on the X-ray structure of GTPγS-bound Gαs (PDB 1AZT). The β2AR from the β2AR-Gs complex structure (PDB 3SN6) was modeled on the top of Gαs to show the position of the β2AR. **b**. The HDX levels of β2AR-bound nucleotide-free Gαs were color-coded on the β2AR-Gs complex (PDB 3SN6). GDP was modeled to show the position of GDP.

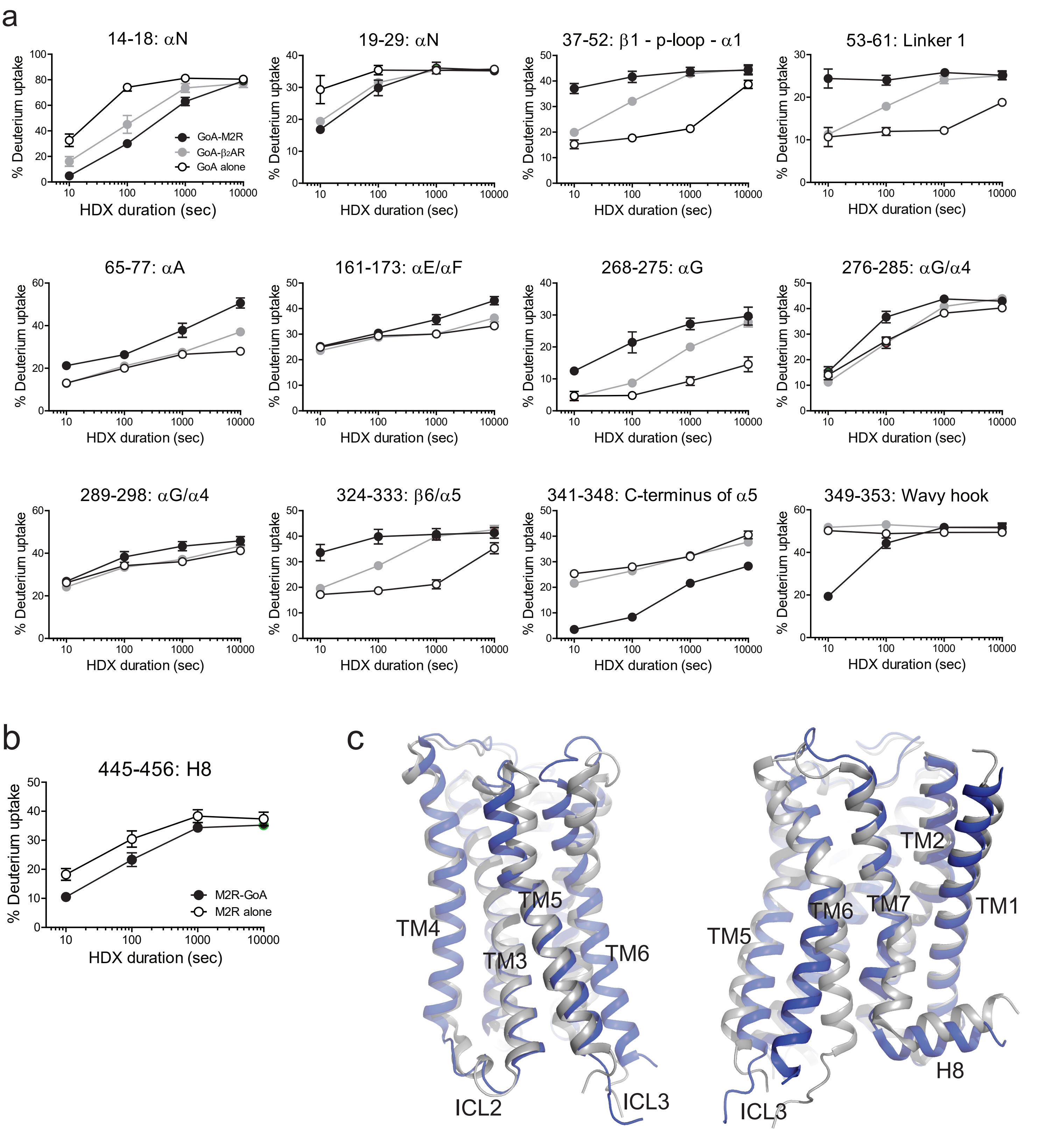

Supplementary Fig. 4. Deuterium uptake plots of peptides from blue- or red-colored regions in figures 1c and 1d (**a**) and 4a (**b**). **a** Deuterium uptake plots of selected peptides from GαoA. **b** Deuterium uptake plot of a peptide from H8 of the M2R. Error bars represent mean ± S.E.M of three independent experiments. **c** Comparison of high-resolution structures of the M2R in G protein-free inactive (grey, PDB 3UON) and GoA-bound (blue, PDB 6OIK) states.

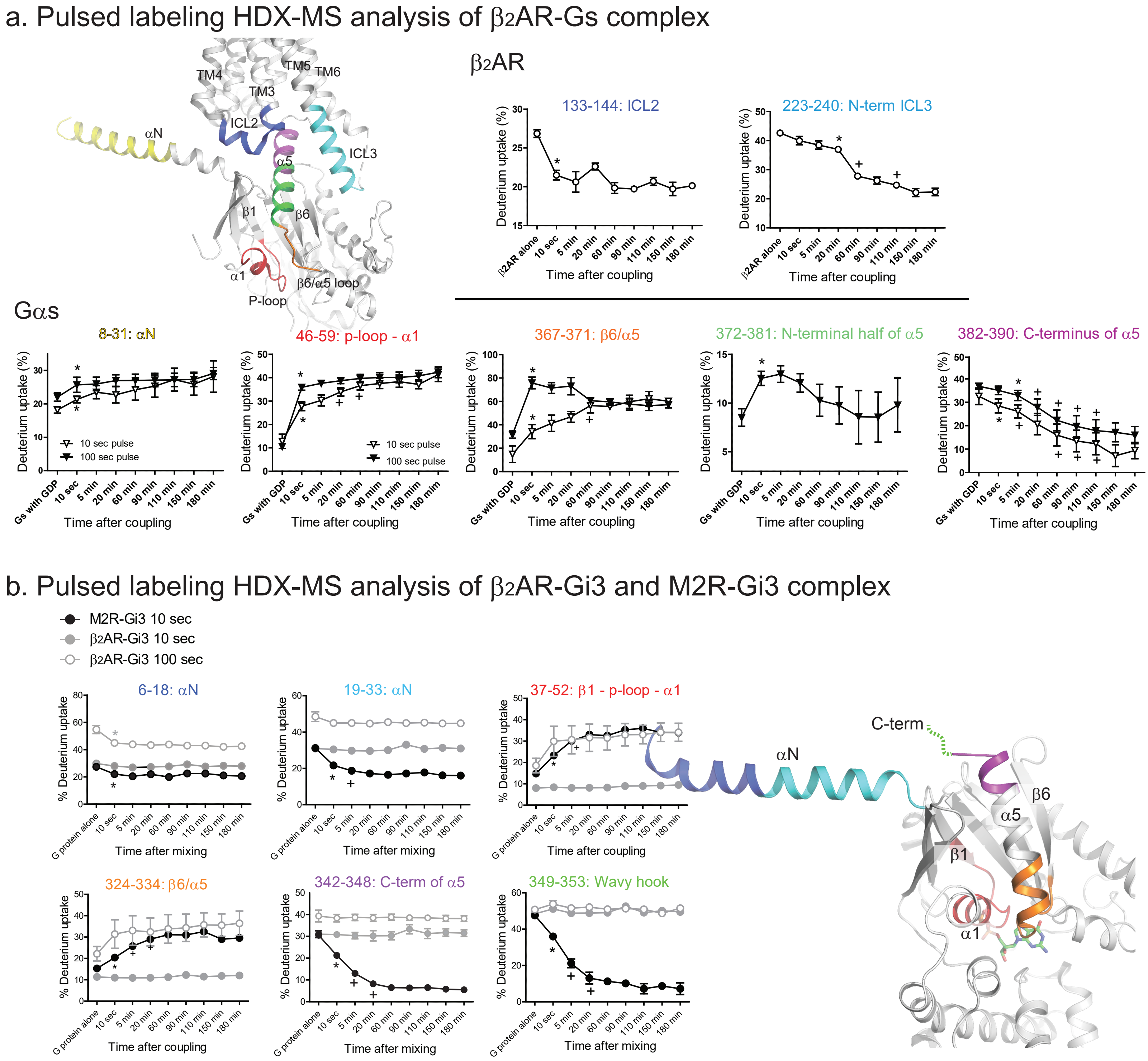

Supplementary Fig. 5. Time-resolved HDX-MS profiles during during β_2AR-Gs coupling (**a**) and M2R-Gi3 and β_2AR-Gi3 coupling (**b**). **a.** The selected analyzed peptides are color-coded on the X-ray crystal structure of β_2AR-Gs complex (PDB 3SN6) and on the titles of the deuterium uptake graphs. The figure was re-produced based on the data previously published by Du *et al.* (19). **b.** The selected analyzed peptides are color-coded on titles of the deuterium uptake graphs and the X-ray crystal structure of Gαi1 (PDB 1GP2).

Statistically significant changes during coupling were analyzed by repeated-measures ANOVA (rANOVA). To compare the HDX levels of two different time points, a two-tailed paired Student's t-test was performed, and $p<0.05$ was considered as statistically significant. * indicates the first time point that showed a statistical difference compared to before coincubation. + indicates the first time point that showed a statistical difference from previously marked (either * or +) time point. Error bars represent mean ± S.E.M of at least three independent experiments (n=3-4). Please note that the data is plotted using a non-linear/non-logarithmic scale.

Supplementary Table 1. List of primers used for mutagenesis

Protein	Construct	Primer
Gαi3	Δ5	F: 5′- CATTAAAAACAACTTATAAGAATGTGGACTTTATTG -3′<br>R: 5′- CAATAAAGTCCACATTCTTATAAGTTGTTTTTAATG -3′
M2R	L129A	F: 5′- GCCTGCCACTTACCCAGTGAAGAGAACCACTAAGATGGCCG -3′<br>R: 5′- GGTAAGTGGCAGGCTTGGTAACACAGAAGTACCTGTCGAAAGAG -3′
β_2AR	F139A	F: 5′- CACCTGCCAAGTACCAGAGCCTGCTGACCAAGAATAAGGC -3′<br>R: 5′- GGTACTTGGCAGGTGAAGTAATGGCAAAGTAGCGATCCACTGC -3′
