# Supplementary material for: Structural mechanism underlying primary and secondary coupling between GPCRs and the Gi/o family
Source: Nat Commun. 2020 Jun 22;11:3160. doi: 10.1038/s41467-020-16975-2 (PMC7308389; doi:10.1038/s41467-020-16975-2)
Supplement: Supplementary file 2 — Description of Additional Supplementary Information [file 41467_2020_16975_MOESM2_ESM.pdf]

Description of Additional Supplementary Files

File Name: Supplementary Data 1
Description:

Sheet 1 (Continuous labeling summary)

Table S1-A. Summary of continuous labeling HDX-MS data of GoA α-subunit upon GPCR coupling. This data corresponds to Supplementary Fig. 4a.

Table S1-B. Summary of continuous labeling HDX-MS data of M2R upon GoA coupling. This data corresponds to Supplementary Fig. 4b.

Table S1-C. Summary of continuous labeling HDX-MS data of β2AR upon GoA coupling.

Sheet 2 (10 sec pulsed labeling summary)

Table S2-A. Summary of 10 sec pulsed labeling HDX-MS data of Gi3 α-subunit upon GPCR coupling. This data corresponds to Supplementary Fig. 5b.

Table S2-B. Summary of 10 sec pulsed labeling HDX-MS data of GoA α-subunit upon GPCR coupling. This data corresponds to Fig. 2

Table S2-C. Summary of 10 sec pulsed labeling HDX-MS data of M2R upon Gi3 and GoA coupling

Table S2-D. Summary of 10 sec pulsed labeling HDX-MS data of β2AR upon Gi3 and GoA coupling. This data corresponds to Fig. 4c

Sheet 3 (100 sec pulsed labeling summary)

Table S3-A. Summary of 100 sec pulsed labeling HDX-MS data of Gi3 α-subunit upon β2AR coupling. This data corresponds to Supplementary Fig

Table S3-B. Summary of 100 sec pulsed labeling HDX-MS data of GoA α-subunit upon β2AR coupling. This data corresponds to Fig. 2

Table S3-C. Summary of 100 sec pulsed labeling HDX-MS data of β2AR upon Gi3 and GoA coupling. This data corresponds to Fig. 4c

Sheet 4 (Continuous labeling HDX)

Table S4-A. Deuterium uptake level of continuous labeling HDX-MS of GoA α-subunit upon GPCR coupling. This data corresponds to Supplementary Fig. 4a

Table S4-B. Deuterium uptake level of continuous labeling HDX-MS of GoA β-subunit upon GPCR coupling

Table S4-C. Deuterium uptake level of continuous labeling HDX-MS of GoA γ-subunit upon GPCR

coupling

Table S4-D. Deuterium uptake level of continuous labeling HDX-MS of M2R upon GoA coupling. This data corresponds to Supplementary Fig. 4b

Table S4-E. Deuterium uptake level of continuous labeling HDX-MS of β2AR upon GoA coupling

Sheet 5 (10s pulsed labeling HDX)

Table S5-A. Deuterium uptake level of 10 sec pulsed labeling HDX-MS of Gi3 α-subunit of upon M2R coupling. This data corresponds to Supplementary Fig. 5b

Table S5-B. Deuterium uptake level of 10 sec pulsed labeling HDX-MS of Gi3 β-subunit upon M2R coupling

Table S5-C. Deuterium uptake level of 10 sec pulsed labeling HDX-MS of Gi3 γ-subunit upon M2R coupling

Table S5-D. Deuterium uptake level of 10 sec pulsed labeling HDX-MS of Gi3 α-subunit upon β2AR coupling. This data corresponds to Supplementary Fig. 5b

Table S5-E. Deuterium uptake level of 10 sec pulsed labeling HDX-MS of Gi3 β-subunit upon β2AR coupling

Table S5-F. Deuterium uptake level of 10 sec pulsed labeling HDX-MS of Gi3 γ-subunit upon β2AR coupling

Table S5-G. Deuterium uptake level of 10 sec pulsed labeling HDX-MS of GoA α-subunit upon M2R coupling. This data corresponds to Fig. 2

Table S5-H. Deuterium uptake level of 10 sec pulsed labeling HDX-MS of GoA β-subunit upon M2R coupling

Table S5-I. Deuterium uptake level of 10 sec pulsed labeling HDX-MS of GoA γ-subunit upon M2R coupling

Table S5-J. Deuterium uptake level of 10 sec pulsed labeling HDX-MS of GoA α-subunit upon β2AR coupling. This data corresponds to Fig. 2

Table S5-K. Deuterium uptake level of 10 sec pulsed labeling HDX-MS of GoA β-subunit upon β2AR coupling

Table S5-L. Deuterium uptake level of 10 sec pulsed labeling HDX-MS of GoA γ-subunit upon β2AR coupling

Table S5-M. Deuterium uptake level of 10 sec pulsed labeling HDX-MS of M2R upon Gi3 coupling

Table S5-N. Deuterium uptake level of 10 sec pulsed labeling HDX-MS of M2R upon GoA coupling

Table S5-O. Deuterium uptake level of 10 sec pulsed labeling HDX-MS of β2AR upon Gi3 coupling. This data corresponds to Fig. 4c

Table S5-P. Deuterium uptake level of 10 sec pulsed labeling HDX-MS of β2AR upon GoA coupling

Sheet 6 (100s pulsed labeling HDX)

Table S6-A. Deuterium uptake level of 100 sec pulsed labeling HDX-MS of α-subunit of Gi3 upon β2AR coupling. This data corresponds to Supplementary Fig. 5b

Table S6-B. Deuterium uptake level of 100 sec pulsed labeling HDX-MS of β-subunit of Gi3 upon β2AR coupling

Table S6-C. Deuterium uptake level of 100 sec pulsed labeling HDX-MS of γ-subunit of Gi3 upon β2AR coupling

Table S6-D. Deuterium uptake level of 100 sec pulsed labeling HDX-MS of α-subunit of GoA upon β2AR coupling. This data corresponds to Fig. 2

Table S6-E. Deuterium uptake level of 100 sec pulsed labeling HDX-MS of β-subunit of GoA upon β2AR coupling

Table S6-F. Deuterium uptake level of 100 sec pulsed labeling HDX-MS of γ-subunit of GoA upon β2AR coupling

Table S6-G. Deuterium uptake level of 100 sec pulsed labeling HDX-MS of β2AR upon Gi3 coupling. This data corresponds to Fig. 4c

Table S6-H. Deuterium uptake level of 100 sec pulsed labeling HDX-MS of β2AR upon GoA coupling.
